# From Life’s Essential 8 to metabolic syndrome: insights from NHANES database and network pharmacology analysis of quercetin

**DOI:** 10.3389/fnut.2024.1452374

**Published:** 2024-10-07

**Authors:** Runze Zhang, Xiuxiu Qiu, Chenming He, Rou Deng, Chenxing Huo, Bangjiang Fang

**Affiliations:** ^1^Department of Emergency, Longhua Hospital, Shanghai University of Traditional Chinese Medicine, Shanghai, China; ^2^Department of Oncology, Longhua Hospital, Shanghai University of Traditional Chinese Medicine, Shanghai, China

**Keywords:** metabolic syndrome, Life’s Essential 8, NHANES, quercetin, network pharmacology, molecular docking

## Abstract

**Background:**

Metabolic syndrome (MetS), or syndrome X, is a collection of metabolic illnesses that affect the body’s health, particularly insulin resistance and obesity. The prevalence of MetS is on the rise, particularly among younger individuals. Quercetin, a natural flavonoid found in many traditional Chinese medicines, can impact various pathways to disrupt the pathological advancement of MetS with few negative effects. The American Heart Association recently introduced a cardiovascular health assessment termed Life’s Essential 8 (LE8), which might impact the treatment of MetS.

**Methods:**

Quercetin targets and their functions in MetS pathways were identified using a network pharmacology method and molecular docking techniques. The study examined quercetin’s direct and indirect interactions with proteins linked to the pathogenic processes of MetS. Data were collected regarding the American Heart Association’s LE8 cardiovascular health indicators, which include health behaviors (diet, physical activity, nicotine exposure, and sleep) and health factors (body mass index, non-high-density lipoprotein cholesterol, blood glucose, and blood pressure). The study assessed the connection between LE8 and the occurrence of MetS, taking into account dietary quercetin consumption as a variable of interest.

**Results:**

The negative correlation between MetS and LE8 indicates that individuals with higher LE8 scores are less likely to develop MetS. Individuals in the fully adjusted highest group (LE8 ≥ 80) demonstrated a 79% lower likelihood of developing MetS than those in the lowest group (OR = 0.21; 95% CI, 0.17–0.26, *p* < 0.0001). Network pharmacology and molecular docking results show that quercetin may exert its therapeutic effects by modulating various biological response processes, including those related to xenobiotic stimuli, bacterial molecules, lipopolysaccharides, and oxidative stimuli. These processes involve key pathways associated with diabetic complications, such as the AGE-RAGE signaling pathway, pathways related to diabetic complications, and pathways involved in lipids and atherosclerosis. Therefore, quercetin may reduce cardiovascular risk, improve glucose-lipid metabolism, and alleviate insulin resistance and other biological processes by influencing multiple aspects of the lipid profile, blood glucose, and insulin resistance, ultimately impacting the links between LE8 score and MetS.

**Conclusion:**

This study discovered that an optimal LE8 score is a marker of adopting a lifestyle of wellness and is connected with a reduced likelihood of developing MetS. Quercetin acts on core targets such as IL6, BCL2, TP53, IL1B, MAPK1, and CCL2, and then plays a therapeutic role in regulating lipid metabolism, anti-inflammation, immunomodulation, autophagy, etc., through the pathways of diabetic complications, lipids, atherosclerosis, etc., and has the characteristics of multi-targets, multi-pathways, and multi-functions in regulating interventions for MetS.

## 1 Introduction

Metabolic syndrome (MetS) is a group of complex illness conditions characterized by metabolic abnormalities (1), a pathological state in which the body’s fats, proteins, and carbohydrates are disrupted. The condition comprises a range of clinical syndromes that significantly affect human health, characterized by symptoms such as obesity, dyslipidemia, and hyperglycemia, and it has become a global public health concern that endangers people’s health. The current definition of MetS is based on four key features: insulin resistance, visceral obesity, atherogenic dyslipidemia, and endothelial dysfunction (2). These factors combine to create a cluster of related illnesses. MetS is caused by intricate interactions including genetic predisposition, dietary choices, and physical activity levels (3), and environmental factors. Comprehensive therapies are needed to address the many components of MetS and its underlying pathophysiological causes due to its intricate character.

Research indicates that quercetin is beneficial for treating MetS and reducing glucose and cholesterol. Quercetin, chemically known as 3,3′,4′,5,7-pentahydroxyflavone, is mostly found as glycosides in plant leaves, flowers, and fruits (4). Traditional Chinese remedies include quercetin (5), including Acanthopanax, *Panax ginseng*, *Ginkgo biloba*, *Morus alba*, and Cuscuta. Quercetin has a variety of biological effects, including antioxidant, anti-inflammatory, antiproliferative, anticarcinogenic, antidiabetic, and antiviral characteristics (6). In terms of food, quercetin is one of the most extensively researched flavonoids. Many fruits and vegetables (e.g., onions, peppers, and asparagus) and other foods naturally contain quercetin (7). Onions are a significant source of quercetin in the human diet, with their dried skin containing a high concentration of quercetin, mostly in the form of aglycones. Studies have shown that quercetin metabolites maintain antioxidant effects in the bloodstream after quercetin is consumed (8). Oral quercetin reduced insulin resistance and controlled hormone levels in overweight individuals with a high cardiovascular disease risk profile in a research (9). Quercetin can enhance the function of brown adipose tissue and promote the conversion of white adipose tissue to brown, which boosts thermogenesis in the body by upregulating coupling proteins. Quercetin has a positive effect on metabolic anomalies in obese mice, suggesting its potential utility in preventing obesity and metabolic disorders (10). The overwhelming evidence indicates that quercetin has beneficial effects on ovarian histomorphology, folliculogenesis, and the luteinization process. Quercetin has been associated with reduced levels of testosterone, luteinizing hormone (LH), and insulin resistance.

The concept of comprehensive lifestyle modification as a therapeutic approach for MetS has been more prominent with research on bioactive compounds like quercetin. The initiative of the American Heart Association, Life’s Essential 8 (LE8), emphasizes eight vital elements to preserve cardiovascular health and avert MetS: diet, physical activity, nicotine exposure, sleep health, body weight, blood lipids, blood glucose, and blood pressure (11). This holistic approach emphasizes the combined impact of lifestyle factors on metabolic health, indicating that adhering to these guidelines may reduce the risk of developing MetS. Quercetin’s action, in conjunction with the principles outlined in LE8, offers a distinct chance to explore integrative strategies for managing MetS.

This study aimed to explore the impact of quercetin on MetS regulation and the association between LE8 and MetS prevalence. The study employed network pharmacology, molecular docking techniques, and data from the U.S. National Health and Nutrition Examination Survey (NHANES). We want to provide novel perspectives on possible synergies for treating MetS, as well as strategies for managing and preventing MetS through lifestyle adjustments.

## 2 Information and methods

### 2.1 Network pharmacology and molecular docking

#### 2.1.1 Target acquisition of quercetin

The TCMSP was used to obtain the target of quercetin using the compound name (chemical name) (12). The UniPort database was utilized to standardize and rectify the projected drug target names to GeneSymbol (13), based on human species and verified genes as screening criteria.

#### 2.1.2 MetS disease target collection

We searched the GeneCards database (Search until 2024.3.13)and the OMIM database (Search until 2024.3.12) for gene targets related to MetS (14, 15), using “metabolic syndrome” as a keyword and specific features of the syndrome, such as “insulin resistance,” “hypertension,” and “hyperlipidemia” as secondary keywords, in addition to “*Homo sapiens*” as a species constraint.

#### 2.1.3 Integration of quercetin and metabolic syndrome disease common targets

The overlap between quercetin and MetS illnesses was identified by analyzing quercetin constituent target and MetS target data using the R 4.3.2 language instrumentation.

#### 2.1.4 Constructing the “active ingredient-target” regulatory network

The regulatory network of quercetin’s active ingredient target for MetS was created by combining the targets of quercetin and MetS in Cytoscape 3.10.1 software.

#### 2.1.5 PPI network building and topology analysis

The overlapping objectives of quercetin and MetS disease were uploaded to the STRING database via R4.3.2 (16). A protein–protein interaction (PPI) network was constructed by filtering the species to human “Homosapiens” with a confidence level exceeding 0.9 and removing solitary targets. The PPI network was imported into Cytoscape 3.10.1 software for topological attribute analysis. The CytoNCA plug-in was utilized to analyze the topological parameters such as degree centrality (DC), closeness centrality (CC), betweenness centrality (BC), eigenvector centrality (EC), and local average connectivity-based method (LAC) to identify key nodes in the network based on network topology concepts. To identify the core network of PPIs and screen the core targets, nodes DC, CC, BC, EC, and LAC have to go above the median of their respective values.

#### 2.1.6 Bioinformatics analysis

We utilized the org.Hs.eg.db package in R 4.3.2 to convert gene names of the intersection targets labeled as “1.1.3” into Entrez IDs. Subsequently, we employed the Cluster Profiler package in R to conduct GO function enrichment and KEGG pathway enrichment analyses. Ultimately, we visualized the results of the enrichment analyses for GO functions and KEGG pathways by generating barplot charts and bubble diagrams.

#### 2.1.7 Molecular docking validation

Molecular docking was performed on the primary target of the medication and its active ingredients. The 3D structures of the active chemical components were retrieved from the PubChem database in SDF format (17), and the small molecular structure of the protein was manipulated using Chem3D 17.0. The crystal structure of the target was acquired from the PDB database and prepared using the AutoDockTools 1.5.6 software (18), which eliminated water molecules and hydrogen atoms. The crystal structures of the target proteins and their active component compounds were converted to PDBQT format using AutodockTools 1.5.6, and molecular docking was performed using Vina software. The results were shown when implementing the PyMOL software.

### 2.2 Data analysis using the NHANES database

#### 2.2.1 Data source

The present study draws on data from the National Health and Nutrition Examination Survey (NHANES), which was performed between 2005 and 2018. NHANES is a useful, publicly available database based in the United States. This massive database has a plethora of information, including a wide range of data points such as demographics, food habits, complete exams, lab tests, and several questionnaires. It also contains a chunk of data labeled as “restricted access,” which requires users to complete a detailed application if they want to study its contents further (19). The Research Ethics Review Board of the National Center for Health Statistics approved the methodology of the study, ensuring that it met the highest standards of ethical research conduct. This endorsement was gained using a solid framework in which all participants supplied written informed consent when they were first recruited. This provided a solid ethical foundation for the investigation.

#### 2.2.2 Study design and population

**Figure 1:**
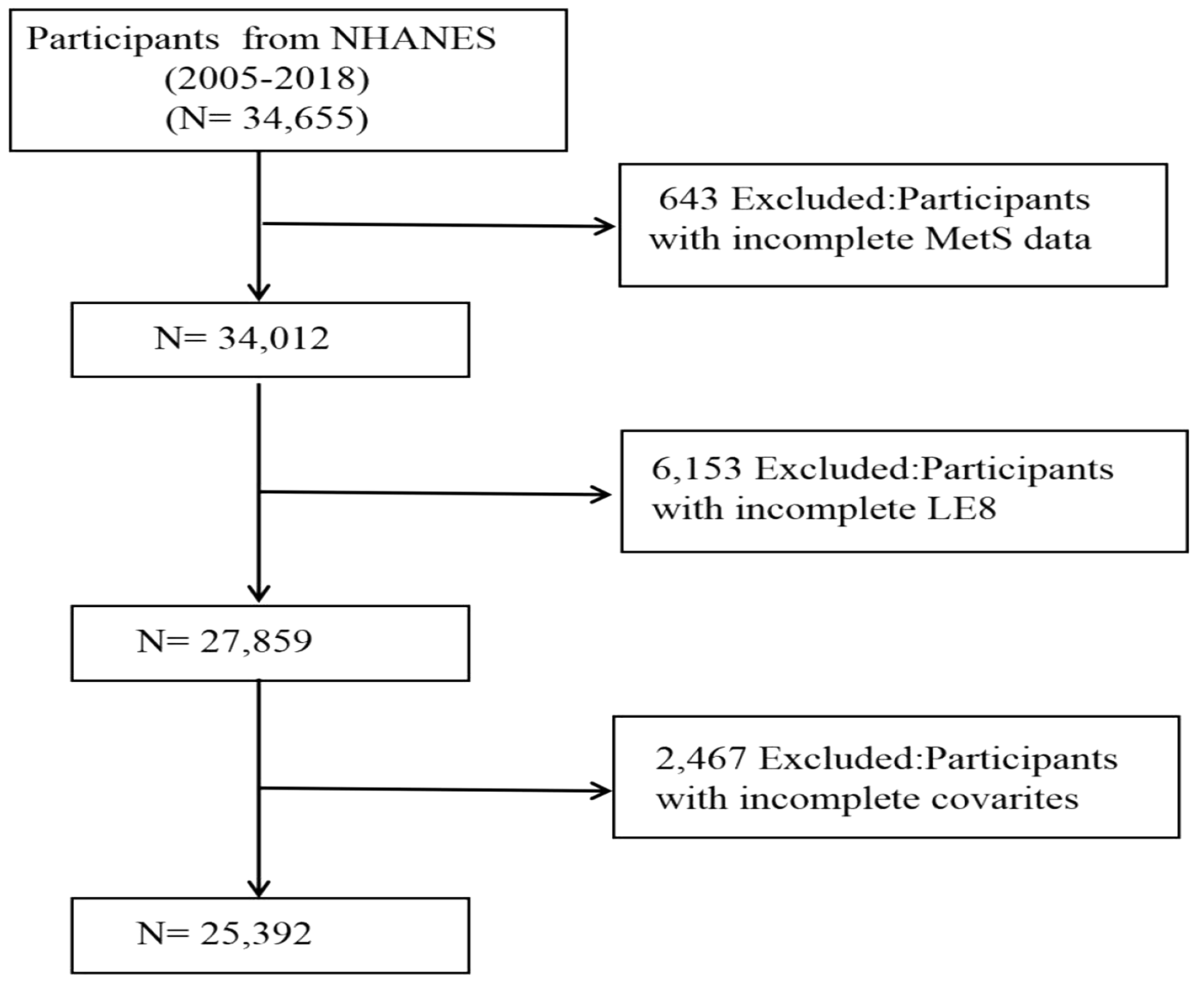
The flow chart of participant selection.

In this research, stringent criteria were applied to eliminate specific groups. Figure 1 depicts the selection process of our study. A total of 34,655 participants aged over 18 years were initially recruited, yet 643 individuals were excluded due to the absence of MetS information, and 6,153 subjects were omitted because of partial LE8 score data. Additionally, 2,467 entries were found to lack detailed information regarding other variables, including PIR, smoking habits, alcohol consumption, past cancer diagnoses, and previous medication use. Following an extensive filtering process, the study ultimately included 25,392 participants.

#### 2.2.3 Covariates

In our study, covariates consisted of several factors previously displayed or assumed to be associated with LE8 or MetS, including age, gender (female and male), ethnicity (Mexican American, Non-Hispanic Black, Non-Hispanic White, or Others), education level (below high school, high school, or above), marital status (married or live together, widowed, divorced or separated, and never married), and family income to poverty ratio (PIR), drinking status (former, heavy, mild, moderate, and never), smoking status (never/former/now), and medical history, comprising hypertension, coronary heart disease (CHD), diabetes, stroke, and cancer were identified through self-reporting (20). The body mass index (BMI) (kg/m^2^) was derived by dividing the weight (kg) of the individual by the square of their height (m).

#### 2.2.4 Assessment of LE8

The American Heart Association has introduced the Life Essential 8 (LE8), an innovative model designed to enhance and preserve cardiovascular wellbeing. This framework employs an integrative method, encompassing eight critical elements: diet, physical activity, nicotine consumption, and sleep quality as the behavioral aspects; and body mass index (BMI), blood lipids, blood glucose, and blood pressure as the health metrics. Each component is evaluated through a novel scoring mechanism, assigning values between 0 and 100 points per factor, as referenced in scholarly articles (19).

In this research, the LE8 scoring system is segmented into three tiers to gage cardiovascular wellness. Scores from 80 to 100 denote a high category, symbolizing peak health conditions. The moderate category, represented by scores from 50 to 79, mirrors a median health state. Scores falling between 0 and 49 are deemed low, highlighting areas requiring substantial enhancement. The dietary aspect is quantitatively analyzed through the Healthy Eating Index (HEI) 2015, providing an intricate evaluation of dietary intake (11). Furthermore, the investigation gathers comprehensive data on physical activity, nicotine intake, sleep quality, and diabetes presence through surveys, while lab tests yield precise details on lipid and glucose concentrations. Conducted by the Mobile Examination Center (MEC), physical assessments yield accurate blood pressure, height, and weight metrics, facilitating the computation of body mass index (BMI) by dividing weight in kilograms by height in meters squared. This thorough approach ensures a detailed and robust appraisal of cardiovascular health via the LE8 model.

#### 2.2.5 Statistical analysis

Given the complex sampling design of NHANES, all analyses in this study were performed with adjustments for sampling weights, clustering, and stratification to ensure nationally representative results. For models that covered NHANES cycles between 2005 and 2018, new weights were calculated by dividing the original 2-year cycle weights by 7, in accordance with NHANES guidelines, to ensure the accurate estimation of national population parameters over the combined study period.

We used the statistical software tool R 4.3.2 to integrate data and make the pictures in this study. Continuous variables were depicted as weighted means along with their standard errors, while categorical variables were represented by frequencies and their associated percentages. To evaluate the odds ratio (OR) and 95% confidence intervals (CI) for the correlation between LE8 and MetS, logistic regression models were employed. To tackle confounding factors, three models with multivariate adjustments were constructed. Model I was simply adjusted for age, ethnicity, and gender; Model II was adjusted for marital status, BMI, education, drinking status, smoking status, and PIR based on Model I; and Model III was adjusted for hypertension, diabetes, stroke, CHD, and cancer history using Model II. The investigation of potential dose–response associations between the LE8 score and MetS was conducted using a restricted cubic spline (RCS) model. Subgroup analysis and interaction testing were used to evaluate how covariates affected the link between LE8 and MetS. For two-sided tests, a *p*-value less than <0.05 was deemed to denote statistical significance.

## 3 Results

### 3.1 Network pharmacology and molecular docking

#### 3.1.1 Quercetin target prediction

After normalization and de-weighting in the Uniprot database, 130 targets were retrieved by searching the TCMSP database for related targets.

#### 3.1.2 MetS disease-related target screening

In total, 18,173 MetS-related targets were acquired from the GeneCards database and 330 from the OMIM database. After summarizing and de-emphasizing the two datasets, a total of 18,173 targets were located.

#### 3.1.3 Integration of quercetin and MetS disease common targets

R 4.3.2 software was used to determine the intersection of quercetin targets and 125 intersecting targets were identified, indicating that quercetin can play a role in MetS via 125 targets such as PTGS1, AR, PPARG, PTGS2, NCOA2, KCNH2, SCN5A, and so on.

#### 3.1.4 Quercetin “active ingredient-target” network for MetS treatment

**Figure 2:**
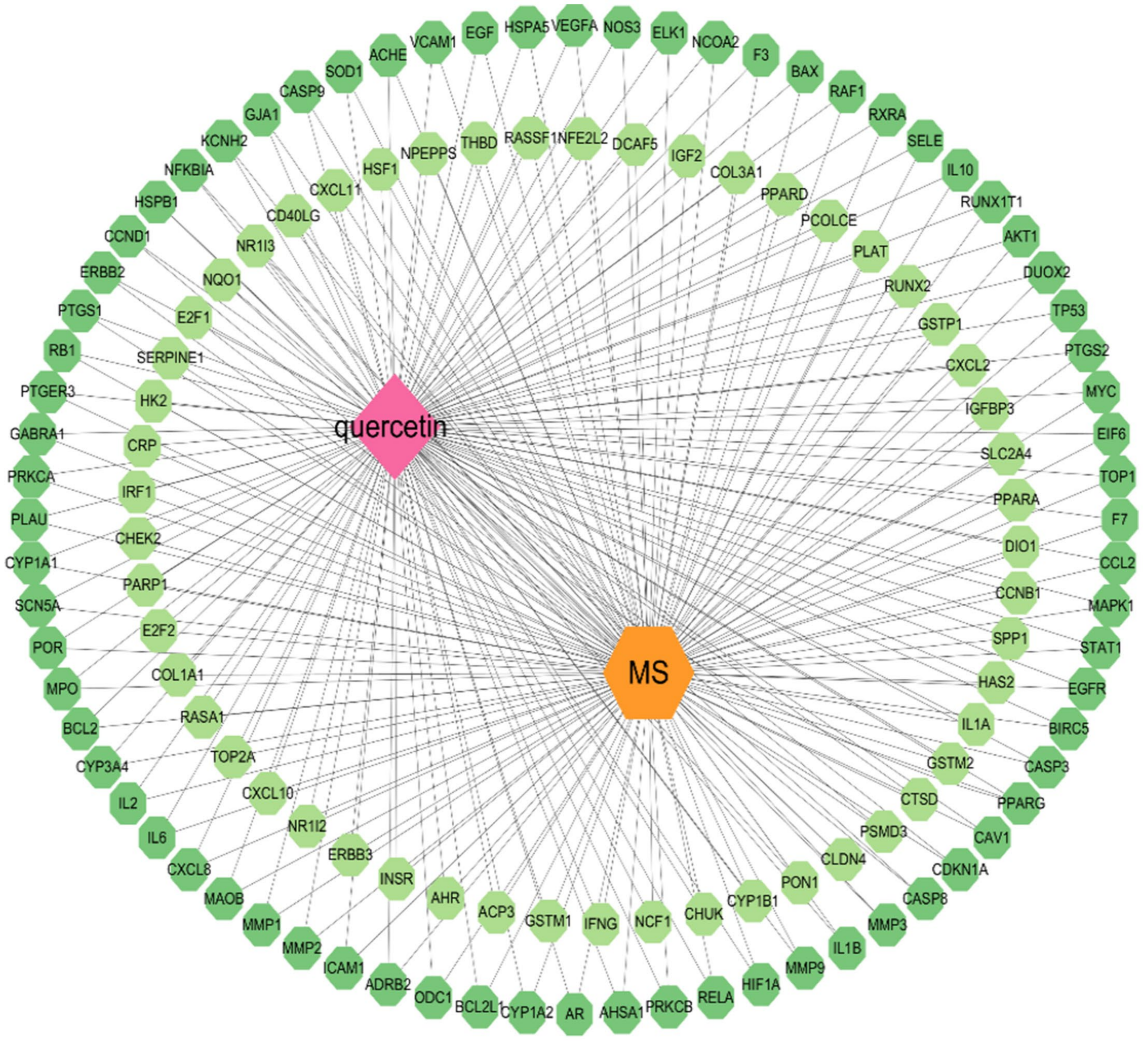
Ingredient–target–disease network.

The mentioned genes were combined, and the data was displayed and analyzed via Cytoscape 3.10.1 software. Nodes indicate substances, illnesses, and genes. The connections between the nodes indicate the complicated interactions among them (see Figure 2).

#### 3.1.5 Topology analysis of PPI network

**Figure 3:**
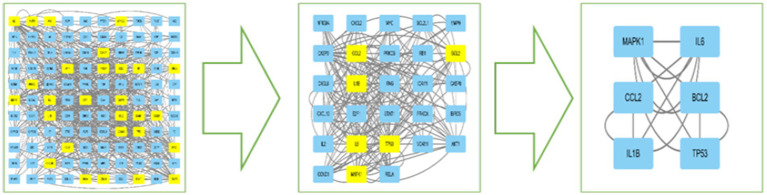
Intersecting targets PPI network core target gene screening flowchart.

The DC, BC, CC, EC, and LAC values of the PPI network were calculated by CytoNCA in Cytoscape 3.10.1 software, and topology analysis was performed two times. The first screening with BC > 76.543, CC > 0.197, DC > 8, EC > 0.055, and LAC >2.545 yielded 29 nodes such as CXCL8, CCL2, IL6, IFNG, IL1B, CXCL2, and CXCL10; and the second screening with BC > 344.488, CC>0.200, DC>22, EC>0.189, and LAC>7.2, finally obtained a core network containing 6 nodes and 14 edges. The 6 nodes were IL6, BCL2, TP53, IL1B, MAPK1, and CCL2, which were the core targets for the treatment of MetS, respectively (see Table 1; Figure 3).

**Table 1:** Topological parameters of the core targets of quercetin therapy for MetS.

Gene	BC	CC	DC	EC	LAC
IL6	1583.435	0.218	38	0.278	10.736
BCL2	401.240	0.201	28	0.196	9.428
TP53	3497.449	0.224	60	0.316	7.866
IL1B	708.255	0.209	30	0.240	11.733
MAPK1	855.107	0.216	28	0.197	7.428
CCL2	1286.871	0.201	24	0.197	12

#### 3.1.6 Bioinformatics analysis

**Figure 4:**
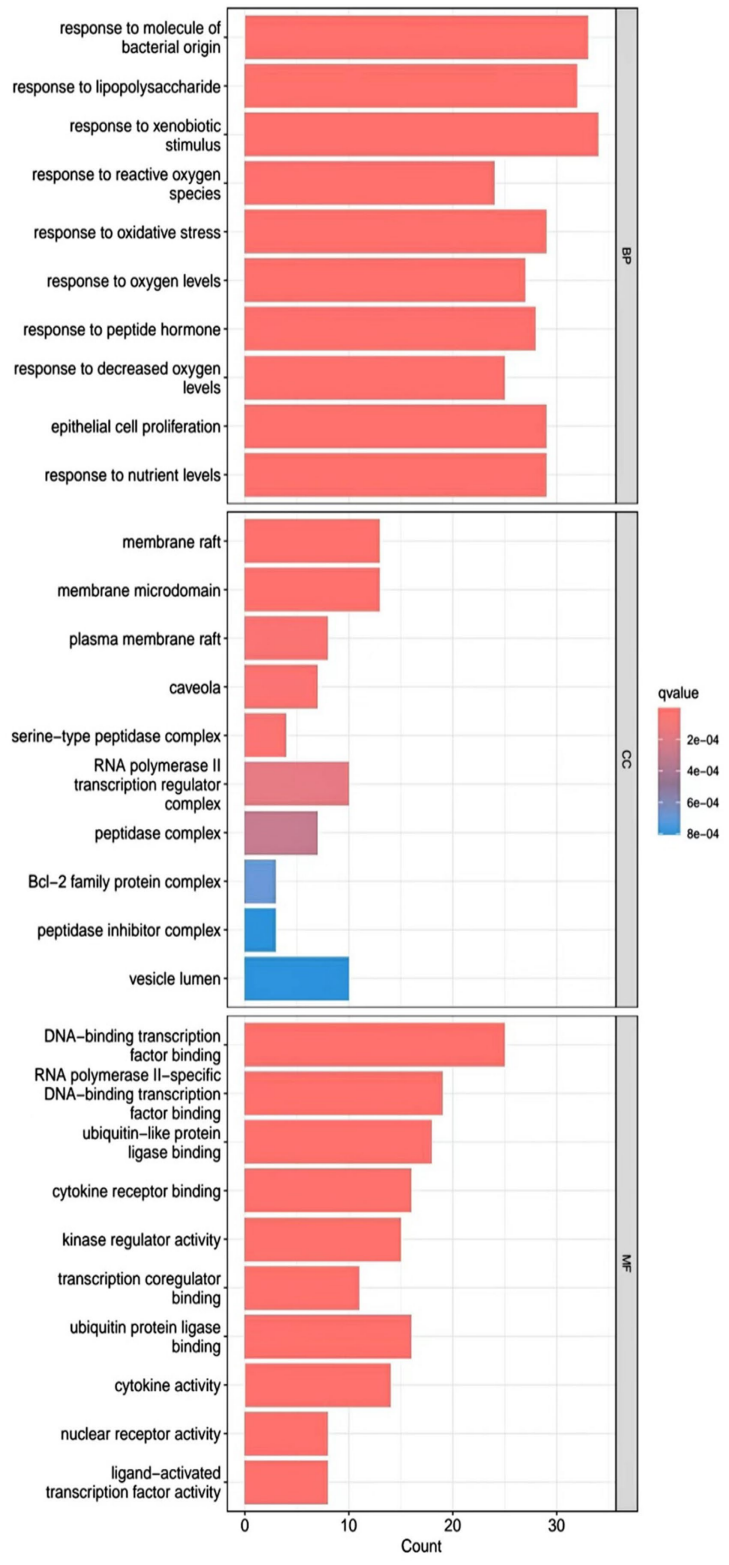
GO enrichment analysis barplot.

**Figure 5:**
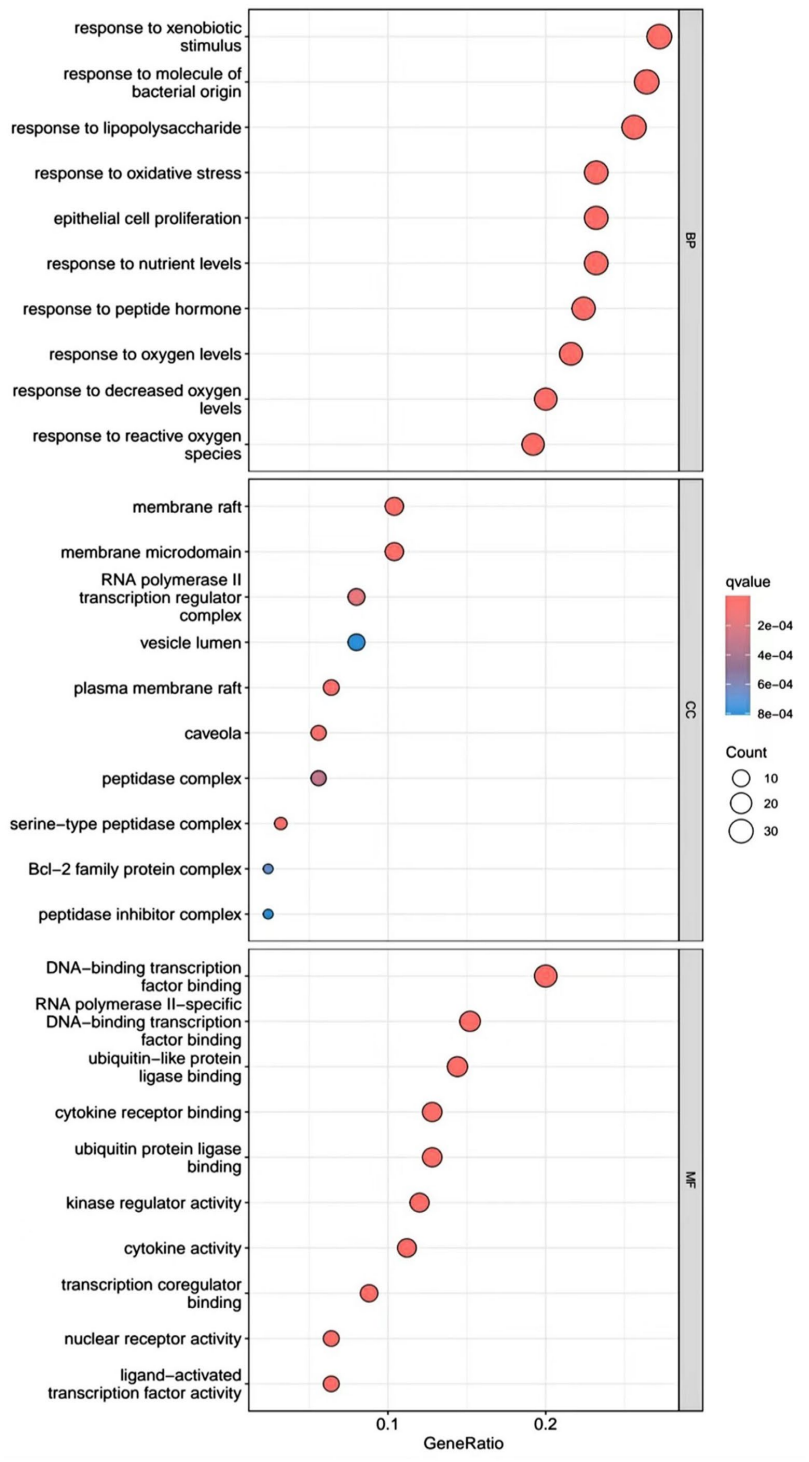
GO enrichment analysis bubble.

**Figure 6:**
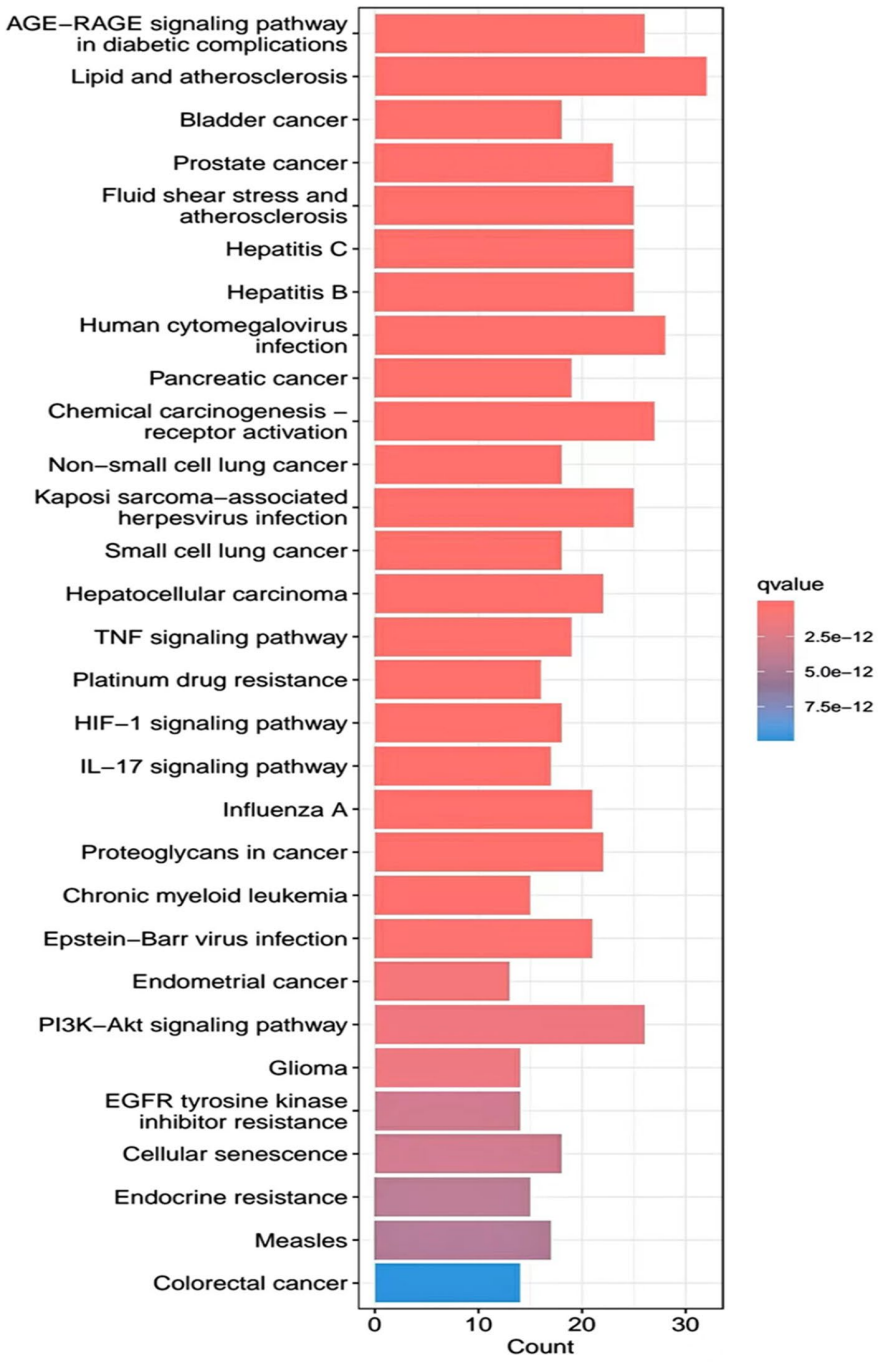
KEGG pathway analysis barplot.

**Figure 7:**
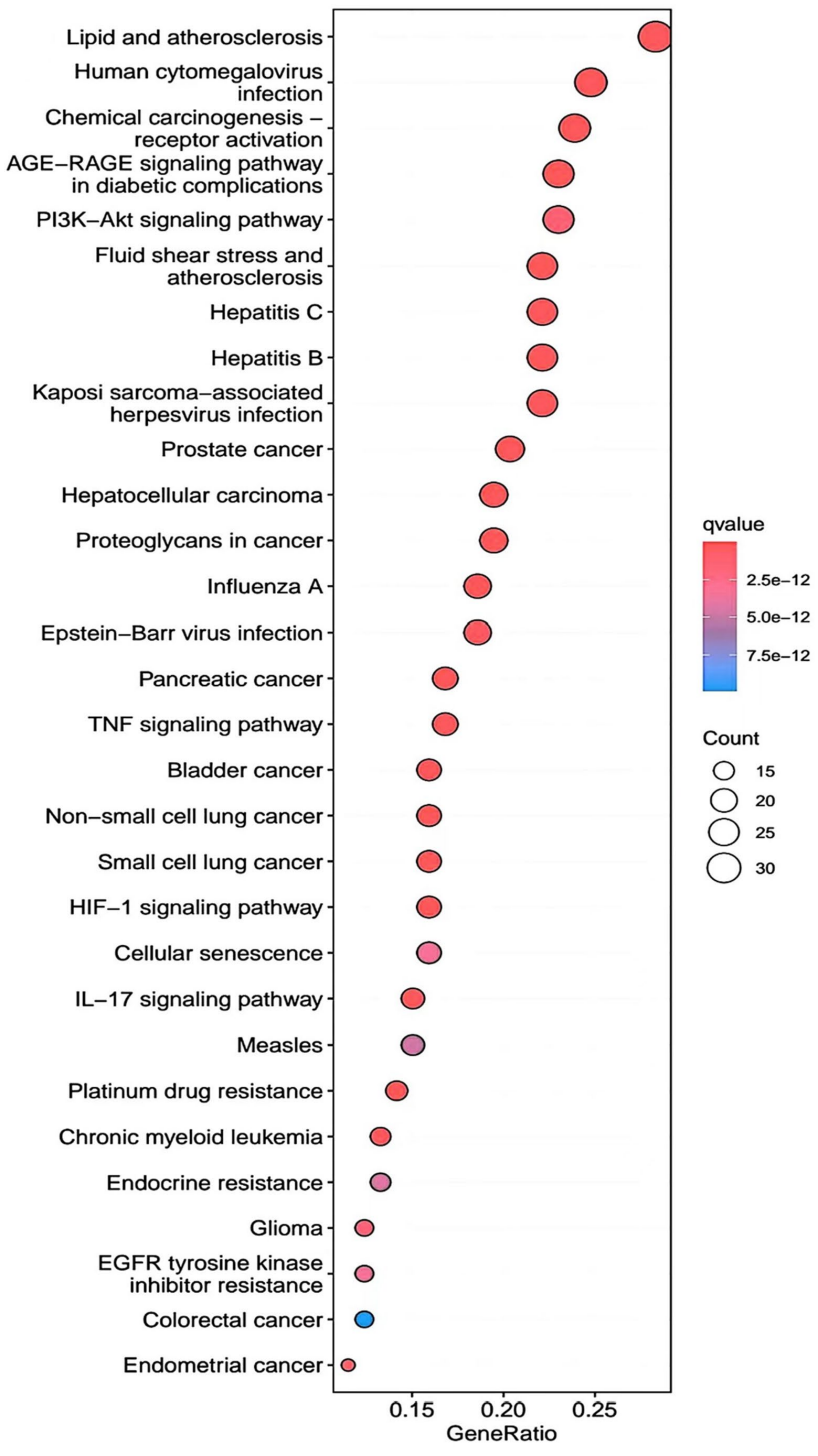
KEGG pathway analysis bubble.

R.4.3.2 software was used to run a GO functional enrichment analysis of 125 common targets, yielding 2,196 entries in total. Among them, the GO-BP enrichment analysis revealed that quercetin may be able to regulate the response to xenobiotic stimuli, the response to bacterial molecules, the response to oxidative stress, epithelial cell proliferation, the response to nutrient levels, and other biological processes that play therapeutic roles. GO-CC’s involvement in illness therapy. According to the GO-CC enrichment study, it is mostly involved in membrane raft, membrane microdomain, RNA polymerase, transcription regular complex, vesicle lumen, and other biological processes. The GO-MF enrichment analysis revealed that the composition of cellular components, such as the vesicle lumen, mainly included DNA-binding transcription factor binding, RNA polymerase-specific DNA-binding transcription factor binding, ubiquitin-like protein ligase binding, cytokine receptor binding, ubiquitin protein ligase binding, and ubiquitin protein ligase binding. According to the KEGG pathway enrichment study, quercetin may be implicated in AGE-RAGE signaling (pathway in diabetic complications), lipid and atherosclerosis via its participation in diabetic complications (see Figures 4–7).

#### 3.1.7 Molecular docking

Generally speaking, the binding energy is usually calculated to evaluate the affinity of the component to the protein target, and a binding energy of less than −5.0 kcal/mol indicates good binding activity between the ligand and the receptor. The binding energy reflects the likelihood of binding between the receptor and the ligand. The lower the binding energy, the higher the affinity between the receptor and ligand and the more stable the conformation. The results showed that the binding energies of quercetin and the core target IL6, BCL2, TP53, IL1B, MAPK1, and CCL2 were all <−5.0 kJ/mol, which indicated that the active ingredient had a better binding activity with the core target. The results are shown in Table 2. In summary, quercetin has a good binding capacity to all six core targets, BCL2, CCL2, IL1B, IL6, MAPK1, and TP53, and may play an important role in the treatment of MS.

**Table 2:** Free energy binding of active ingredients to core targets.

Molecule name	Target gene	Combined energy (kcal/mol)	PDB ID
Quercetin	BCL2	−7.4	4lxd
Quercetin	CCL2	−5.8	3ifd
Quercetin	IL1B	−7.9	2nvh
Quercetin	IL6	−7.2	1alu
Quercetin	MAPK1	−7.6	8aoj
Quercetin	TP53	−8.1	2g3r

**Figure 8:**
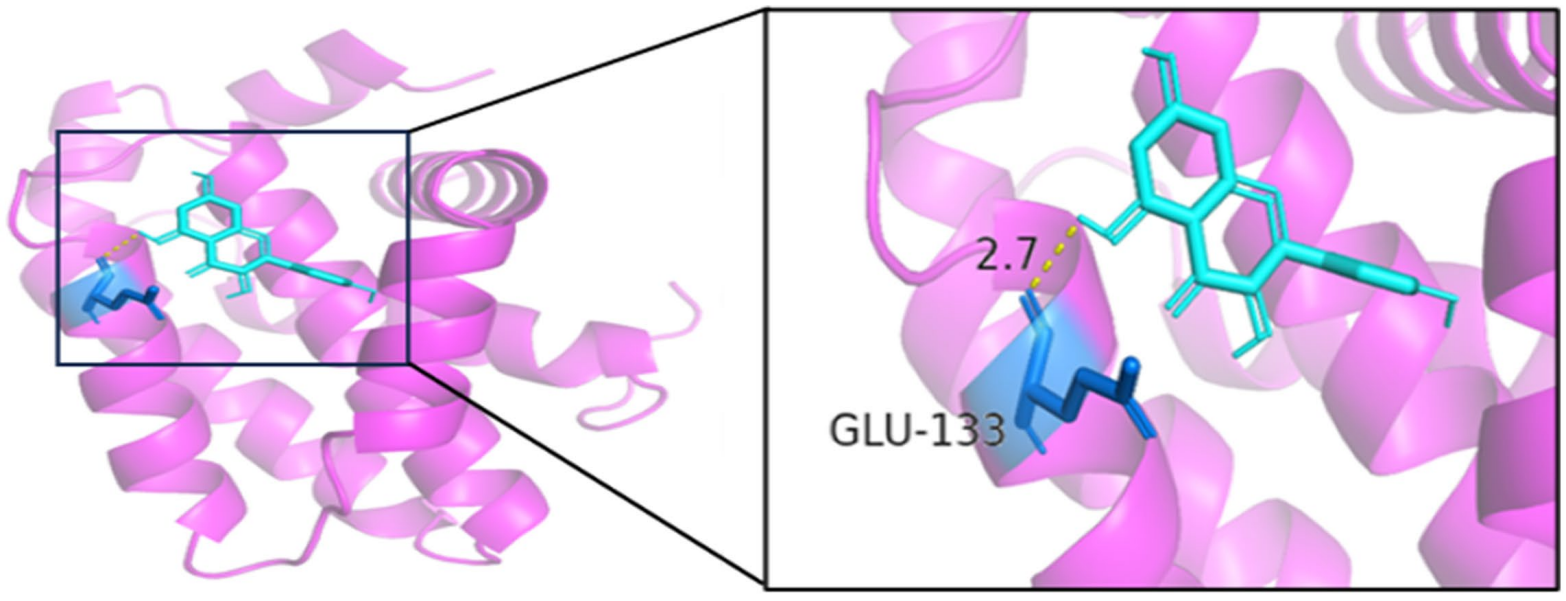
BCL2-quercetin.

**Figure 9:**
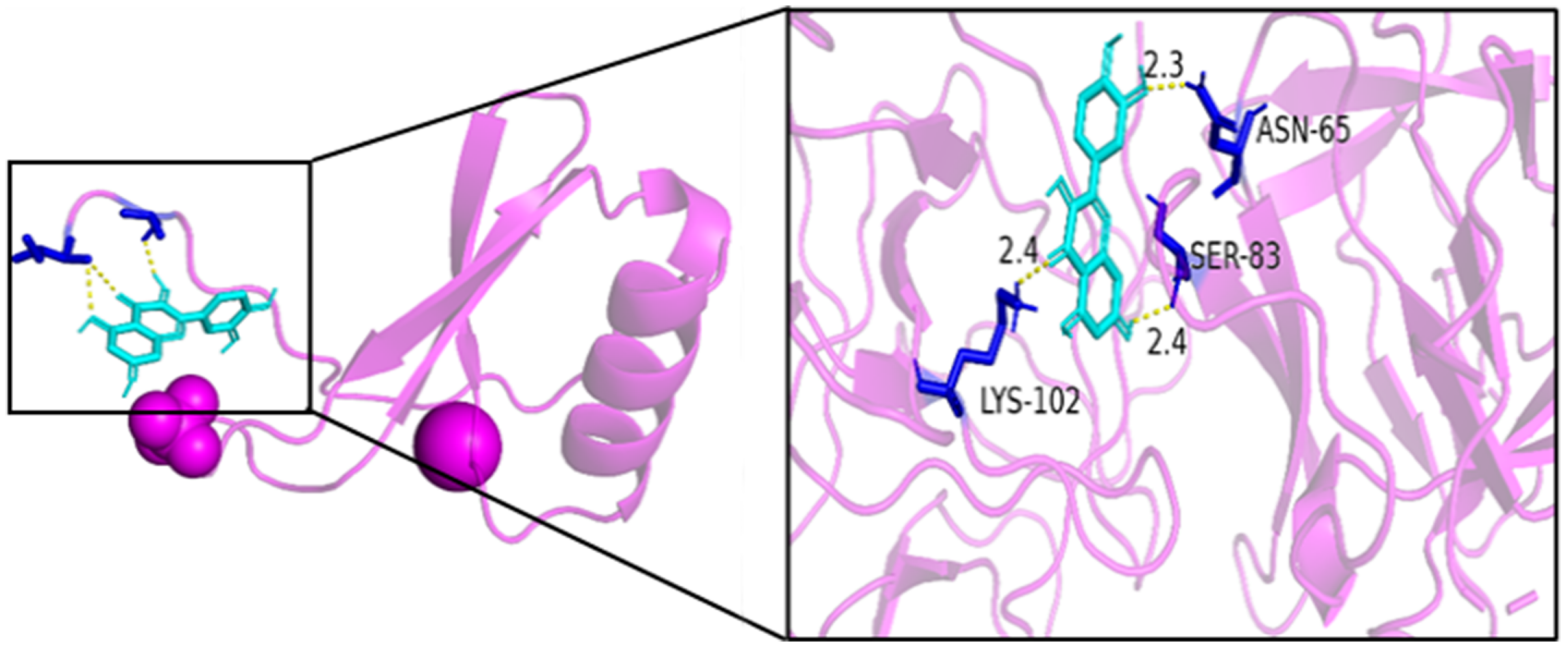
CCL2-quercetin.

**Figure 10:**
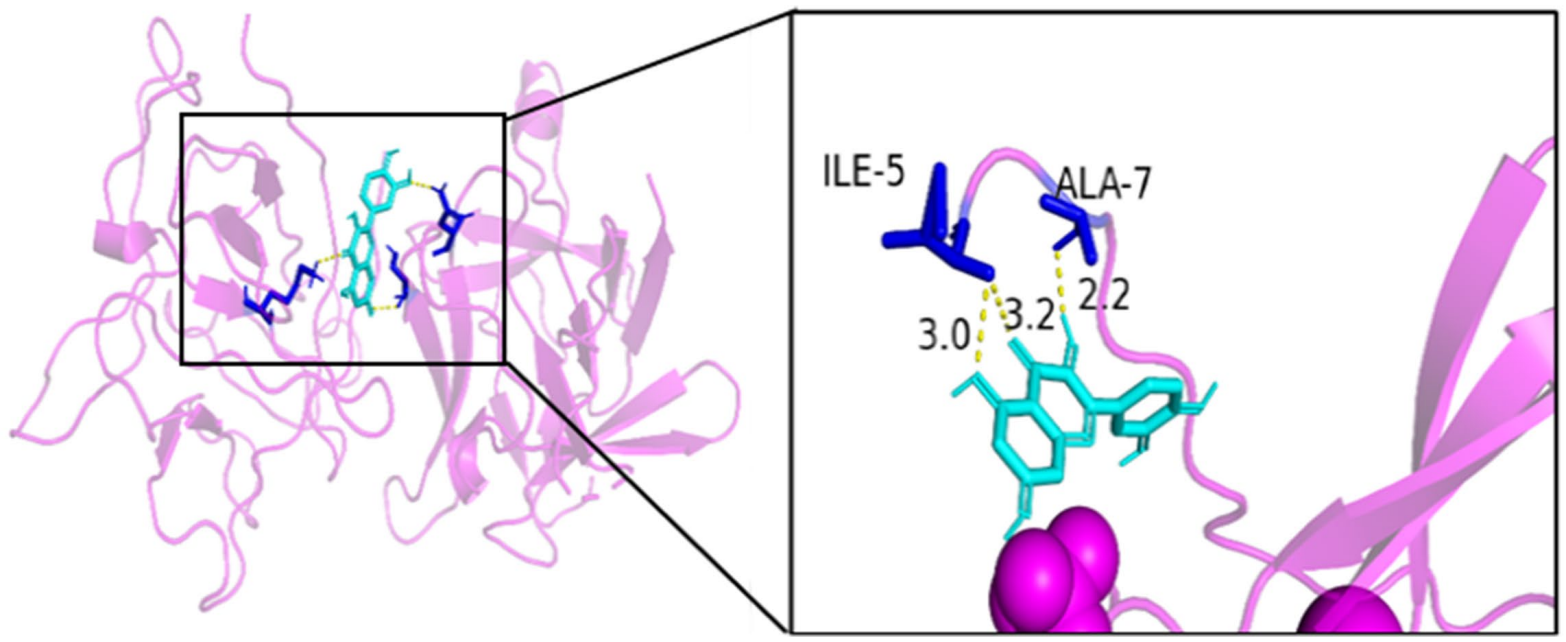
IL1B-quercetin.

**Figure 11:**
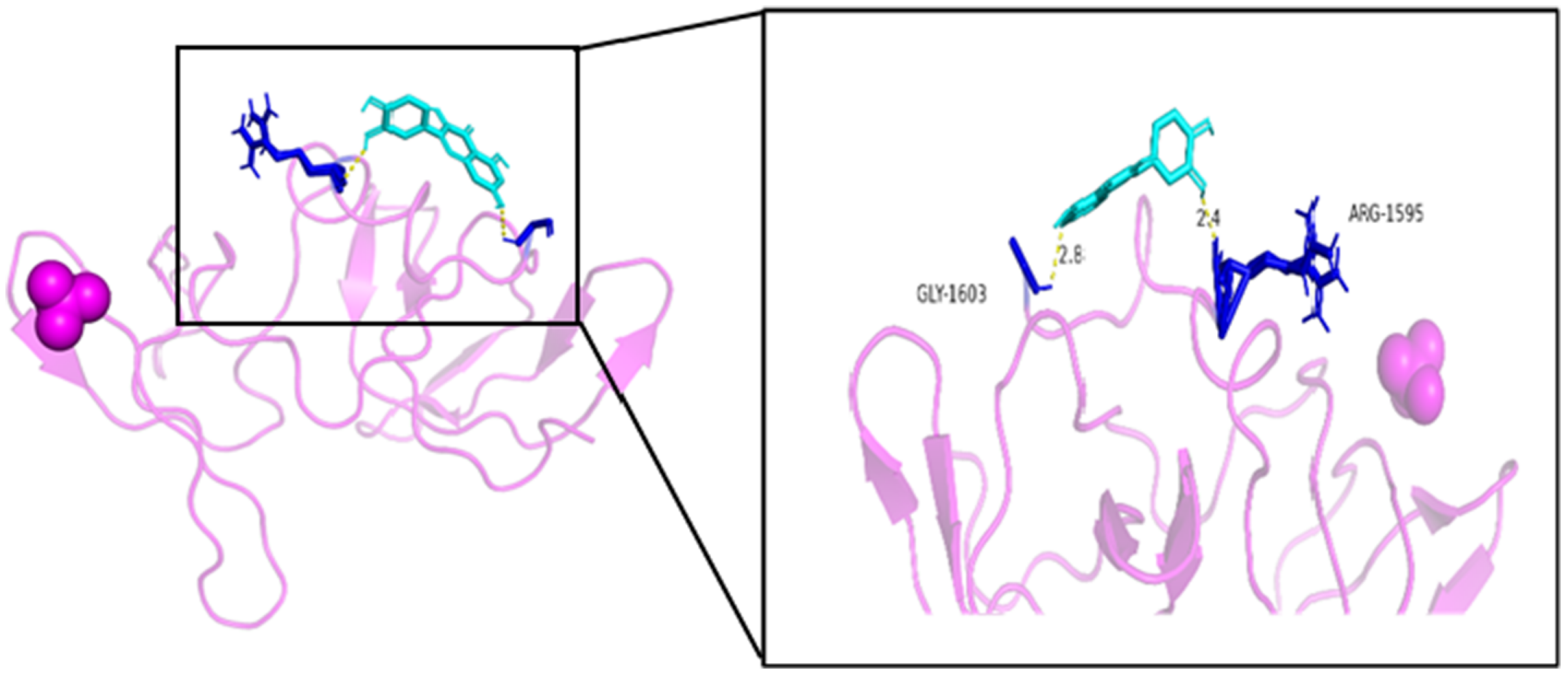
IL6-quercetin.

**Figure 12:**
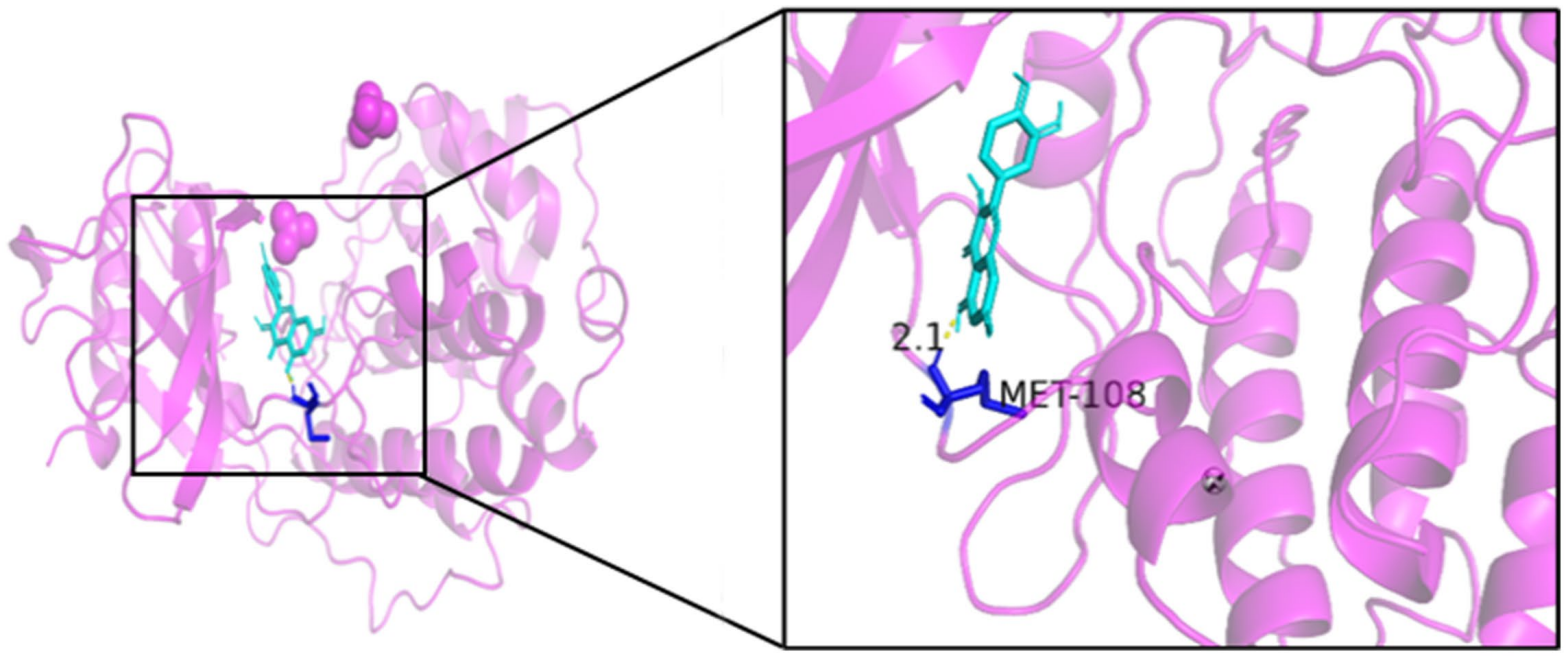
MAPK1-quercetin.

**Figure 13:**
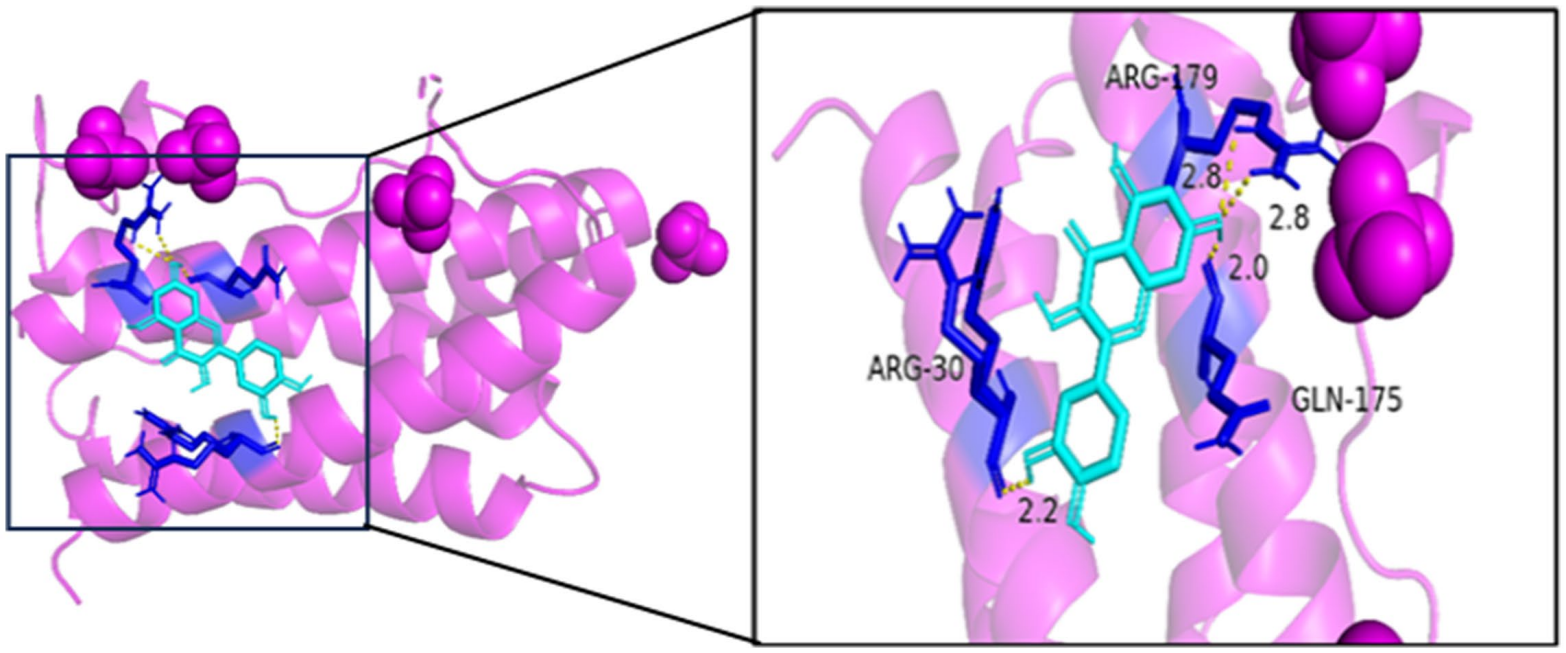
TP53-quercetin.

As seen in Figures 8–13, the potential binding site of quercetin with BCL2 was GLU-133, with CCL2 were ASN-65, SER-83, and LYS-102, with IL1B were ILE-5 and ALA-7, with IL6 was MET-108, with MAPK1 were ARG-30, ARG-179, and GLN-175, and the potential binding sites with TP53 were GLY-1603 and ARG-1595, indicating that quercetin could play a regulatory role on the core targets.

#### 3.1.8 Baseline characteristics of participants

Table 3 presents an overview of the participants’ characteristics. The research had a total of 25,392 participants, with an average age of 47.83 ± 0.25 years. Of these individuals, 48.33% were men, 51.67% were women, and 31.69% had MetS. The average and variability of LE8 were 67.97 ± 0.24. Significant differences were observed in many characteristics between groups without MetS and those with MetS, including age, ethnicity, marital status, BMI, education level, drinking status, smoking status, PIR, hypertension, diabetes, stroke, CHD, and cancer history (all *p* < 0.05).

**Table 3:** Baseline characteristics of participants.

Variables	Overall	Without MetS	With MetS	*p-*value
		*N* = 1,6,759	*N* = 8,633	
Age (years)	47.83 ± 0.25	44.83 ± 0.28	54.31 ± 0.27	< 0.0001
Gender, %				0.08
Male	12,441 (48.33)	8,484 (48.85)	3,957 (47.21)	
Female	12,951 (51.67)	8,275 (51.15)	4,676 (52.79)	
Ethnicity/Race, %				< 0.0001
Mexican	3,674 (7.44)	2,258 (7.18)	1,416 (8.01)	
White	5,359 (10.39)	3,762 (11.13)	1,597 (8.80)	
Black	11,693 (70.83)	7,463 (69.56)	4,230 (73.55)	
Other	4,666 (11.34)	3,276 (12.12)	1,390 (9.64)	
Education level, %				< 0.0001
High school or above	23,200 (95.71)	15,508 (96.25)	7,692 (94.53)	
Below high school	2,192 (4.29)	1,251 (3.75)	941 (5.47)	
PIR	3.09 ± 0.03	3.15 ± 0.04	2.96 ± 0.04	< 0.0001
LE8	67.97 ± 0.24	72.41 ± 0.24	58.39 ± 0.24	< 0.0001
BMI	29.14 ± 0.09	27.18 ± 0.08	33.38 ± 0.10	< 0.0001
Marriage, %				< 0.0001
Married or live together	15,369 (64.73)	10,008 (63.64)	5,361 (67.08)	
Widowed	1937 (5.51)	980 (4.17)	957 (8.38)	
Divorced or separated	3,643 (12.70)	2,193 (11.72)	1,450 (14.79)	
Never married	4,443 (17.07)	3,578 (20.46)	865 (9.75)	
Drinking status, %				< 0.0001
Former	4,256 (13.57)	2,308 (10.97)	1948 (19.18)	
Heavy	4,928 (20.42)	3,511 (21.86)	1,417 (17.30)	
Mild	8,813 (37.80)	5,967 (38.06)	2,846 (37.24)	
Moderate	3,990 (17.83)	2,887 (19.39)	1,103 (14.45)	
Never	3,405 (10.39)	2086 (9.71)	1,319 (11.84)	
Smoking status, %				< 0.0001
Former	6,398 (25.57)	3,768 (23.03)	2,630 (31.06)	
Never	13,914 (55.19)	9,542 (57.42)	4,372 (50.38)	
Now	5,080 (19.24)	3,449 (19.56)	1,631(18.56)	
Hypertension, %				< 0.0001
No	14,430 (61.86)	11,723 (74.64)	2,707 (34.32)	
Yes	10,960 (38.14)	5,034 (25.36)	5,926 (65.68)	
Cancer history, %				< 0.0001
No	22,817 (89.46)	15,327 (91.25)	7,490 (85.81)	
Yes	2,560 (10.47)	1,425 (8.75)	1,135 (14.19)	
Diabetes, %				< 0.0001
DM	4,700 (13.93)	1,164 (4.61)	3,536 (34.03)	
IFG	1,123 (4.52)	440 (2.36)	683 (9.19)	
IGT	1,009 (3.62)	602 (3.10)	407 (4.76)	
No	18,560 (77.92)	14,553 (89.94)	4,007 (52.02)	
Stroke, %				< 0.0001
No	24,366 (96.98)	16,311 (98.10)	8,055 (94.88)	
Yes	1,000 (2.92)	435 (1.90)	565 (5.12)	
CHD, %				< 0.0001
No	24,324 (96.51)	16,369 (98.25)	7,955 (92.74)	
Yes	1,068 (3.49)	390 (1.75)	678 (7.26)	

BMI, body mass index; CHD, coronary heart disease; PIR, the ratio of family income to poverty. Continuous variables are presented as the mean ± standard deviation,and categorical variables showed with *n* (%). *p* < 0.05 means statistically significant.

#### 3.1.9 The association between LE8 and MetS

The results showed that the risk of MetS decreased as the LE8 score increased, as shown in Table 2. Both Model I (OR = 0.93, 95% CI: 0.92–0.93, *p* < 0.0001) and Model II (OR = 0.94; 95% CI, 0.94–0.94, *p* < 0.0001) showed a statistically significant connection. Model III revealed a strong correlation between the LE8 score and MetS, with an OR of 0.96 and a 95% CI of 0.96–0.97 (*p* < 0.0001).

When LE8 scores were divided into groups, the moderate (50 ≤ LE8 < 80) and high (LE8 ≥ 80) LE8 score groups had decreased probabilities of MetS across all models compared to the low LE8 score group (LE8 < 50), which served as the reference category. Compared to the lowest group, persons in the fully adjusted highest group showed a 79% reduction in the chance of getting MetS (OR = 0.21; 95% CI, 0.17–0.26, *p* < 0.0001). Participants in the intermediate group had a substantially decreased risk of MetS compared to the lowest group (OR = 0.61;95% CI, 0.54–0.69, *p* < 0.0001). The findings suggest a strong inverse association between LE scores and the likelihood of MetS, indicating that higher LE8 scores are significantly associated with reduced odds of MetS, a relationship that is consistent across multiple analytical models (Table 4).

**Table 4:** The association between LE8 and MetS.

Exposure	Model I^a^ (OR, 95%CI, *p*-value)	Model II^b^ (OR, 95% CI, *p*-value)	Model III^c^ (OR, 95% CI, *p*-value)
LE8 score	0.93 (0.92,0.93) <0.0001	0.94 (0.94,0.94) <0.0001	0.96 (0.96,0.97) <0.0001
LE8 score (groups)			
Low (LE8 < 50)	Ref	Ref	Ref
Moderate (50 ≤ LE8 < 80)	0.26 (0.23,0.28) < 0.0001	0.38 (0.34, 0.42) < 0.0001	0.61 (0.54, 0.69) < 0.0001
High (LE8 ≥ 80)	0.03 (0.02,0.04) < 0.0001	0.09 (0.07, 0.11) < 0.0001	0.21 (0.17, 0.26) < 0.0001

LE8, Life’s Essential 8; OR, odds ratio; CI, confidence interval.

^aModel I was adjusted for age, ethnicity, and gender.^

^bModel II was adjusted for age, ethnicity, gender, marriage, BMI, education level, drinking status, smoking status, and PIR.^

^cModel III was adjusted for age, ethnicity, gender, marriage, BMI, education level, drinking status, smoking status, PIR, hypertension, cancer history, stroke, and CHD.^

#### 3.1.10 Analysis of restricted cubic spline regression

**Figure 14:**
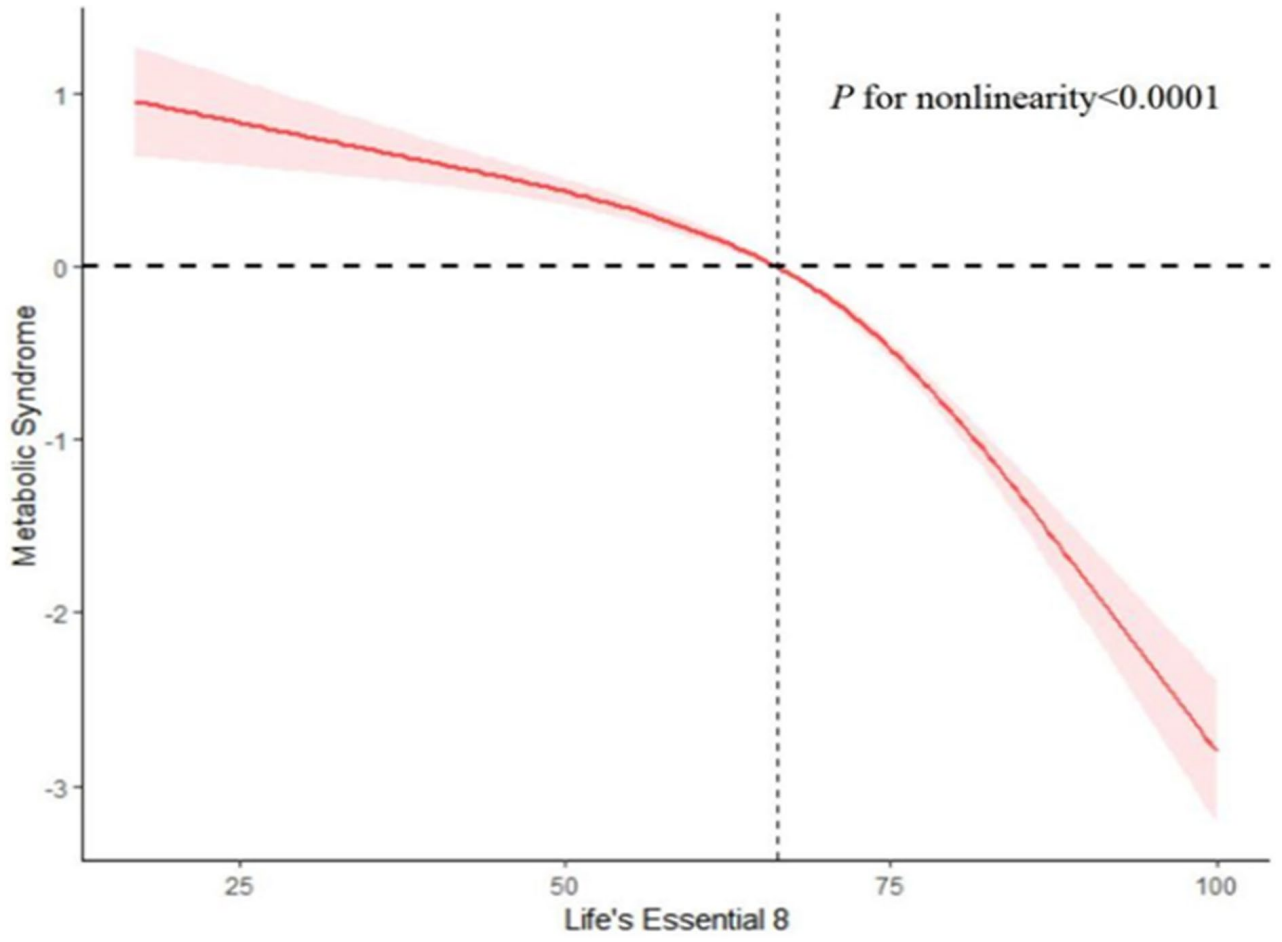
Analysis of restricted cubic spline regression. Restricted cubic spline analysis of hazard ratios (HRs) and 95% confidence intervals (CIs) depicts the association between Life’s Essential 8 (LE8 scores) and metabolic syndrome (MetS).

After accounting for multiple covariates, we identified a significant non-linear relationship between LE8 and MetS in RCS regression (*p* < 0.0001, Figure 14). As the LE8 score increases, MetS decreases significantly.

#### 3.1.11 Subgroup analysis

**Figure 15:**
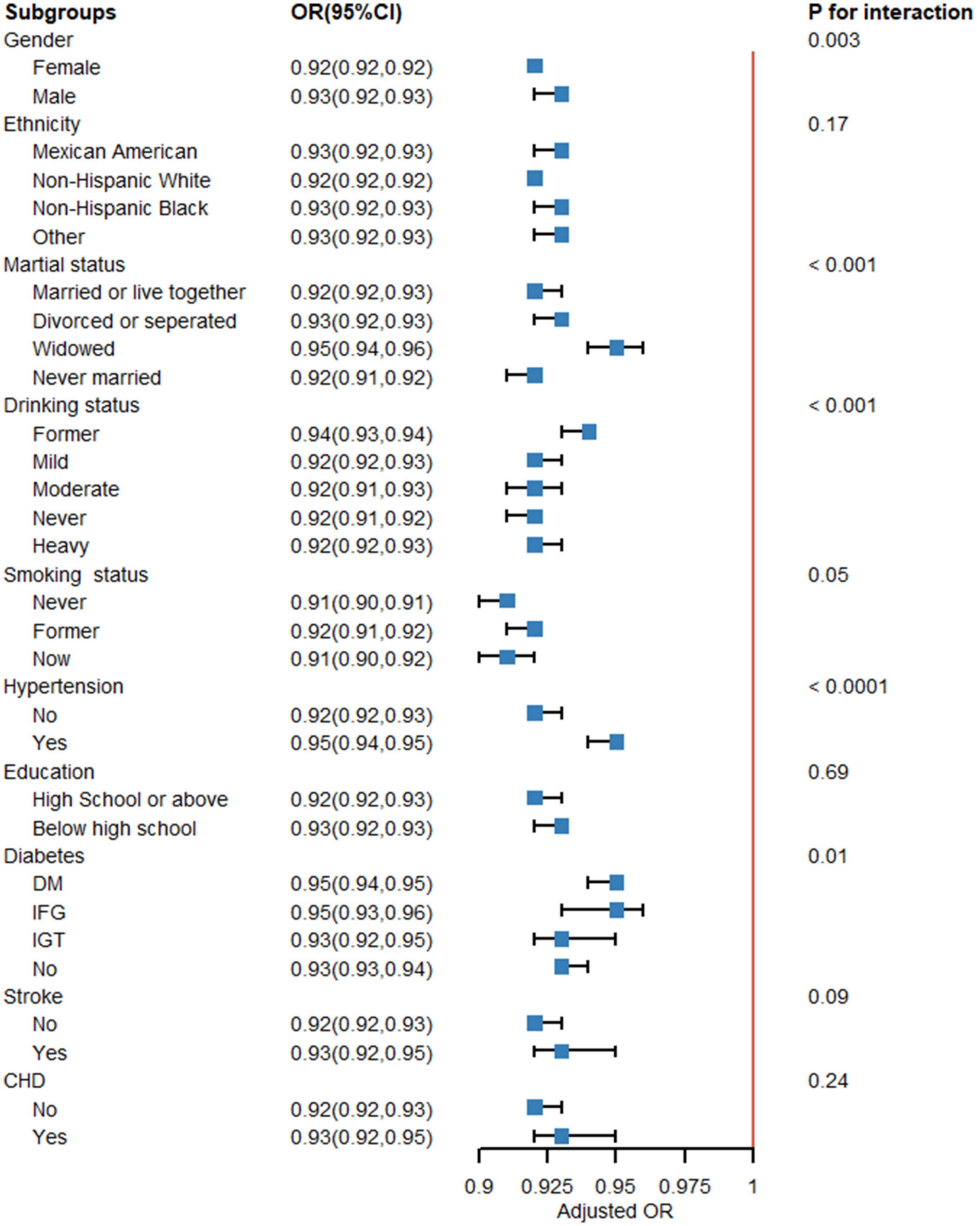
Subgroup analysis. Subgroup analysis and interaction analysis between Life’s Essential 8 (LE8 scores) and metabolic syndrome (MetS). A weighted logic regression model was utilized to conduct the subgroup analyses. Interaction analysis utilizes the likelihood ratio test.

Figure 15 provides further evidence that the LE8 score and MetS are negatively correlated, as indicated by the subgroup analysis findings. There were no significant variations in correlations between LE8 and MetS for race, education, stroke, and CHD, indicating that these variables did not significantly depend on the positive connection (all interactions > 0.05). Gender, marital status, drinking status, hypertension, and diabetes can impact the favorable connection between LE8 and MetS (interaction *p* < 0.05).

## 4 Discussion

This study integrated network pharmacology insights with NHANES epidemiology data. The results highlight quercetin’s ability to alter key pathways associated with MetS and the broader influence of a comprehensive lifestyle on metabolic wellbeing.

### 4.1 Association of quercetin with core genes

In inflammatory responses, quercetin-induced miR369-3p in dendritic cells downregulates C/EBP-*β*, thereby leading to reduced production of TNF- *α* and IL6 (21), which has potential therapeutic implications in modulating the inflammatory cascade in the chronic inflammatory response. A study showed that carbon nanotubes induce immunotoxicity (22), and quercetin can ameliorate its induced immunotoxicity, inflammation, and oxidative response through mechanisms such as decreasing TNF-α and IL6 concentrations, and mRNA levels.

In apoptosis, in a study in which quercetin protected against cisplatin-induced ovarian toxicity in rats, quercetin significantly inhibited the expression of NFjb, IL-6, and Bcl2, which resulted in protective and anti-apoptotic activities (23). Quercetin exhibited significant antioxidant effects by preventing MDA accumulation and glutathione depletion in the ovary (24). In addition, quercetin modulates Bax/BCl2 in the mouse cerebral cortex and hippocampus and reduces activated cytochrome c, caspase-3 activity, and PARP-1 cleavage rate (25), thereby rescuing mitochondrial apoptotic pathway and neuronal degeneration.

In human immune metabolism, quercetin induced higher expression levels of SOD2, IL6, and BAX in AMD cell hybrids (26), which improved AMD cell metabolism.

In disease therapy, a new quercetin glycoside can show potent antitumor activity against colorectal and hepatocellular carcinoma via TP53 (27). Quercetin inhibits IL-8 and CCL2 expression in airway epithelial cells through the PI3K/Ak signaling pathway, and pretreatment with quercetin and PI 3- kinase inhibitors reduces TNF-*α*-induced IL8 and CCL2 (28) expression, respectively, in human airway epithelial cells. It can inhibit allergen sensitization-induced MCP-1 expression and airway hyperresponsiveness *in vivo*, and appears to be effective for the treatment of asthma (29).

### 4.2 Quercetin’s role in metabolic syndrome treatment

Quercetin modulates many processes related to the development of MetS, such as insulin sensitivity, lipid metabolism, and inflammation, as shown by network pharmacology research (30–32). The results align with previous studies indicating that quercetin’s antioxidant and anti-inflammatory properties might be crucial in enhancing metabolic markers and reducing risk factors associated with MetS (33). Our research indicates that quercetin may enhance the benefits of a nutritious lifestyle, such as diet and physical activity programs, by interacting synergistically with other components.

Abdominal obesity can be explained by the accumulation of excessive fatty acids and triglycerides in the liver and other organs (34), which results in organ dysfunction. Quercetin treatment can enhance abdominal obesity generated by monosodium glutamate by restoring imbalances in gut bacteria, which lead to damage in the hypothalamus and anomalies in retinol metabolism (35). Quercetin’s potential to reduce obesity is associated with a decrease in fat accumulation and cell death by blocking the production of enzymes like MAPK and ACC. Increased expression of MAPK and its substrate, acetyl coenzyme A carboxylase, induces cell death and decreases the activation of extracellularly regulated and stress-activated protein kinases. Quercetin diminishes adipogenesis by stimulating the MAPK signaling pathway (36).

Chronic high-fat, high-energy diets can damage the area of the brain that synthesizes acetylcholinesterase (37). Quercetin can reduce the effect of hyperlipidemia on memory impairment by affecting the cholinergic system in the nervous system, thereby increasing the activity of acetylcholinesterase (38). NF-kB activation contributes to an elevation in inflammatory factors, while quercetin suppresses NF-kB function and the production of inflammatory factors in pancreatic cells. ATP, a biological chemical pathway linked with damage, is generated in the vicinity of lysed cells and in high concentrations during infections. Quercetin can decrease hyperlipidemia and inflammatory processes by reducing ATP and ADP levels in the extracellular environment (39).

Quercetin’s advantageous effect on blood glucose levels may be due to many mechanisms, including boosting glucose uptake through GLUT4 and boosting glucokinase activity, promoting liver absorption of glucose, and suppressing liver glycogenolysis and gluconeogenesis (40), or alleviating oxidative stress and pancreatic *β*-cell damage, which can play a positive role in the control of diabetes mellitus.Akt, a serine protein kinase, can control adipogenesis and lipid metabolism via influencing LDL receptors. Quercetin has been apparent to stimulate the SIRT1 gene by increasing NAD+ levels. This regulates Akt function by enhancing deacetylation, shields β-cells from cytokine-induced negative consequences, and decreases diabetic nephropathy. Specifically, quercetin lowers glucose levels in diabetic rats’ blood via boosting Akt and glycogen synthase kinase 3 phosphorylation (41). In addition, SIRT1 activation has been associated with prolonged cellular lifespan, improved metabolic health, and suppression of inflammatory responses, Quercetin can inhibit the NF kappa B pathway through activation of SIRT1, which can ameliorate obesity-induced BAT inflammation (42) and alleviate the effects of iron metamorphosis in order to prevent aging-associated diseases. It also protects NP cells from apoptosis and prevents ECM degeneration via the SIRT1-autophagy pathway (43). In conclusion, quercetin is believed to activate SIRT1, thereby increasing its deacetylation activity, inhibiting oxidative stress, attenuating inflammatory responses, and restoring mitochondrial dysfunction, making it a promising therapeutic target for the treatment of aging-related diseases (44). Catechins and quercetin, present in Amaranthus, improved hyperglycemia and insulin sensitivity through the activation of AKT2 and AMPK (45), increasing the expression of GLUT4, AKT2, and AMPK alpha 2, whose levels are reduced under diabetic conditions. Quercetin, which preferentially activates the AMPK pathway and accordingly stimulates PI3K/Akt signaling, significantly induces GLUT4 translocation in mouse skeletal muscle (46). A study showed that quercetin could enhance GLUT4 expression by activating AMPK in a non-insulin-dependent mechanism (47).

### 4.3 The impact of Life’s Essential 8 on metabolic health

Our examination of NHANES data shows a significant link between a higher LE8 score and a lower risk of MetS. This finding emphasizes the need for a holistic lifestyle strategy that includes nutrition, physical exercise, sleep health, and other aspects of controlling MetS. It is noteworthy that the LE8 contributes to a comprehensive strategy that targets the many risk factors for MetS, emphasizing the notion that no one intervention is likely to be as successful as a complex lifestyle change (48).

### 4.4 Integrating quercetin’s role with lifestyle modifications

The combination of quercetin’s role with the principles of LE8 provides a viable option for MetS treatment. Our findings indicate that quercetin, along with other bioactive substances, may complement lifestyle prograMetS targeted at lowering MetS risk. This alternative approach is consistent with the rising acknowledgment of the importance of dietary patterns, rather than individual nutrients, in affecting health outcomes. Future treatments might benefit from integrating flavonoid-rich foods as part of a larger nutritional approach (49), within the framework of the holistic lifestyle guidelines indicated in LE8.

### 4.5 Limitations and future directions

While our research provides valuable insights, it is not without limitations. Due to the cross-sectional nature of the NHANES data, we were unable to establish causality, only confirming the association between LE8 and MetS. Although this study primarily examines the overall relationship between LE8 and MetS, investigating the individual components of LE8 could yield a more detailed understanding of how each factor uniquely influences health outcomes. Future research should focus on this to explore the underlying mechanisms of these metrics and their effects on metabolic health. Additionally, while network pharmacology offers valuable predictions about new targets and pathways, its findings must be validated by empirical studies. Future research should incorporate longitudinal and interventional designs to establish causal relationships and examine the combined effects of quercetin and lifestyle changes on MetS.

## 5 Conclusion

Abdominal obesity, lipid metabolism abnormalities, and insulin resistance are among the diagnostic criteria for MetS, which is one of the eight components of life. According to the results of network pharmacology and molecular docking, quercetin may intervene in the MetS by participating in the AGE-RAGE signaling pathway in diabetic complications, lipids, and atherosclerosis as the main regulatory pathways; and according to the analysis of NHANES data, hypertension, diabetes mellitus, and body mass index may influence the correlation between LE8 and MetS, and it can be obtained that there is an inverse. As a result, we hypothesized that quercetin might regress MetS through food, blood glucose, and blood lipid control, therefore influencing the LE8 MetS score, which gives research ideas and references for quercetin’s future development and clinical use.

## Data Availability

The data in the article were obtained from open databases, and the search keywords/login numbers have been specified. The public can access them by visiting the following website. NHANES: https://www.cdc.gov/nchs/nhanes/index.htm; TCMSP: http://tcmspw.com/tcmsp; Uniprot: http://www.uniprot.org/uploadlists/; Gnencards: https://www.genecards.org/; Omim: https://www.omim.org/; String: https://string-db.org/; Pubchem: https://pubchem.ncbi.nlm.nih.gov/; PDB: https://www.rcsb.org/.
